# 1602. Determinants of Early Virologic Failure Among HIV patients on First-line Antiretroviral Therapy

**DOI:** 10.1093/ofid/ofad500.1437

**Published:** 2023-11-27

**Authors:** Diana G Ramos, Arthur Dessi E Roman

**Affiliations:** Research Institute for Tropical Medicine, Las Pinas, National Capital Region, Philippines; Research Institute for Tropical Medicine, Las Pinas, National Capital Region, Philippines

## Abstract

**Background:**

The initiation of anti-retroviral therapy (ART) effectively restores immune function and reduces HIV-related morbidity and mortality. However, virologic failure while on ART may lead to increased morbidity and compromised quality of life. The aim of this study was to determine the prevalence and determinants of early virologic failure (VF) among HIV patients on first-line ART enrolled at the Research Institute for Tropical Medicine, Philippines.

**Methods:**

A retrospective cohort study was conducted involving treatment-naive HIV patients who were started on ART during the period of January 2004 – December 2018. Early virologic failure was defined as HIV-1 viral load of > 1,000 copies / ml of plasma blood taken within 6 months or more of first-line ART. Data regarding patients’ sociodemographic profile, baseline clinical characteristics, and treatment-related information were collected through database review. Data were analyzed using STATA 15.0. Descriptive statistics, cross-tabulations, and univariate and multivariate logistic regressions were utilized. P < 0.05 was used to determine clinical significance.

**Results:**

Among the 1900 subjects included in this study, 1839 were males (96.79 %). The mean age at ART initiation was 30.36 years (SD 6.89) with a baseline CD4 count of 232.30 cells / mm^3^ (SD 204.19). The average time from ART initiation to the first VL determination was 4 years (SD 28.62). A total of 72 (3.79 %) subjects were found to have virologic failure. Multivariate logistic regression showed that patients with age 15-20 years were 5-6 times more likely to have VF (1 / adjusted OR 0.18 = 5.55, p-value of 0.024) while patients with baseline CD4 count of < 50 cells/mm^3^ were 3 times (1 / adjusted OR 0.35 = 2.86, p-value < 0.01) more likely to have virologic failure.

**Conclusion:**

The prevalence of early virologic failure among HIV patients on first-line anti-retroviral therapy is 3.79 %, relatively lower compared to other published data. Early virologic failure was most likely to occur among patients 15-20 years old and/or those with baseline CD4 count below 50 cells/mm^3^. Presence of these factors should be used as a marker for intensified adherence counselling especially in the absence of reliable or readily available viral load monitoring.

**Disclosures:**

**All Authors**: No reported disclosures

